# A novel benzoxazinone derivative YLT-LL-11 inhibits diffuse large B-cell lymphoma growth via inducing cell cycle arrest and apoptosis

**DOI:** 10.1042/BSR20190828

**Published:** 2019-10-11

**Authors:** Cuiting Peng, Changzhen Sun, Ningyu Wang, Yuanmin He, Jixiang Xu, Yongqiong Deng, Lanyang Gao, Jianqiao Zhong, Xia Xiong, Li Liu

**Affiliations:** 1 Department of dermatology, The Affiliated Hospital of Southwest Medical University, Luzhou, China; 2 Drug Research Center of Integrated Traditional Chinese and Western Medicine, Affiliated Traditional Chinese Medicine Hospital, Southwest Medical University, Luzhou, China; 3 Department of Obstetrics & Gynecology, West China Second University Hospital, Sichuan University, Chengdu, China; 4 School of Life Science and Engineering, Southwest Jiaotong University, Chengdu, China; 5 Department of Science and Technology, The affiliated hospital of Southwest Medical University, Luzhou, China

**Keywords:** apoptosis, BRD4, cell cycle, Diffuse large B cell lymphoma

## Abstract

Diffuse large B-cell lymphoma (DLBCL) is a clinically aggressive B-cell non-Hodgkin’s lymphoma (NHL) with high treatment difficulty and high relapse rate. The bromodomain and extra-terminal (BET) proteins play significant roles in supporting the transcription of known DLBCL oncogene MYC, which provides a way for the development of targeted therapeutic agents to address this kind of malignant tumor. Here, we reported a novel benzoxazinone derivative YLT-LL-11 as potential BRD4 inhibitor and further investigated the biological activities against DLBCL. The results suggested that YLT-LL-11 inhibited cell growth against a panel of human hematopoietic malignancies cell lines in a dose- and time-dependent manner. In addition, flow cytometry and Western blotting assays showed that YLT-LL-11 inhibited the proliferation of a DLBCL cell line OCI-LY10 via inducing G0/G1 cell cycle arrest with regulation of the cyclin-dependent kinases (CDKs) expression. Furthermore, YLT-LL-11 facilitated OCI-LY10 cell apoptosis by up-regulation of pro-apoptotic protein BAX and down-regulation of anti-apoptotic protein Bcl-2. Taken together, these results revealed that BRD4 inhibitor YLT-LL-11 can down-regulate growth-associated transcription factors MYC in DLBCL thus resulted in cell growth inhibition and apoptosis.

## Introduction

Diffuse large B-cell lymphoma (DLBCL) is the most prevalent B-cell non-Hodgkin’s lymphoma (NHL) accounting for more than one third of those new diagnoses [1]. Though it can be cured in more than 50% of cases [2], over 30% of DLBCL patients do not respond to the current first-line treatment regimens or relapse with drug resistant partly due to the considerable heterogeneity of DLBCL [3–5]. Autologous stem cell transplantation (ASCT) brings hope to those chemoresponsive patients with relapsed or refractory DLBCL, but only a small number of patients would benefit from this method since a proportion of relapsing patients are not transplant eligible [6–8]. On the other hand, the treatment outcomes for those of approximately 20–30% of DLBCL cases overexpressing c-Myc and Bcl-2 remains poor [9]. Thus, it highlights the urgent need for novel therapeutic approaches for these patients.

Bromodomain and extra-terminal domain (BET) proteins are a family of epigenetic adaptors which play a crucial role in the transcriptional regulatory programs [10,11]. As a member of BET family, BRD4 can regulate gene expression via recruiting of transcriptional regulatory complexes to acetylated chromatin and maintain or facilitate oncogenic transcription by interacting directly with transcription factors [12–14]. Various studies have verified that the mis-regulated expression of transcription factors is important for cancer cell proliferation and survival in malignant hematopoietic [15–17]. BRD4 inhibition can down-regulate growth-associated transcription factors MYC and E2F, resulting in antiproliferative effects across DLBCL subtypes, which proved BRD4 to be a particularly appealing target in DLBCL [18–21].

We recently described the discovery of a series of benzoxazinone derivatives as BET bromodomain inhibitors and evaluated their inhibitory activities against BRD4 (BD1) based on affinity assays [22]. Those N-(3-oxo-3,4-dihydro-2H-benzo[b] [1,4] oxazin-7-yl) benzenesulfonamide derivatives displayed favorable affinity with BRD4 (BD1) and potent inhibitory activities against several hematologic malignancies at low-micromolar concentrations. Further optimization on this chemotype obtained YLT-LL-11 with better BRD4 affinity, with an IC_50_ of 1 μM, and favorable IC_50_ against malignant hematopoietic cells MV-4-11, OCI-LY10, and RAMOS. We further investigated the potential mechanism underlying the sensitivity of DLBCL cells to BRD4 inhibitors using OCI-LY10 cells. These results revealed that the BRD4 inhibitor YLT-LL-11 decreased OCI-LY10 cell viability by down-regulating the transcription and expression of c-Myc. Moreover, YLT-LL-11 inhibited tumor cell proliferation via inducing G0/G1 cell cycle arrest and facilitated tumor cell apoptosis. Thus, the present study further verified that targeting of BRD4 by small-molecule inhibitors could be an effective means to inhibit DLBCL tumor growth and/or survival.

## Materials and methods

### Materials

Chemistry reagents of analytical grade were purchased from JUHUI Chemical Factory, Chengdu, Sichuan, PR China and were used without further purification. 3-(4, 5-Dimethylthiazol-2-yl)-2, 5-diphenyltetrazolium bromide (MTT), dimethyl sulfoxide (DMSO), propidium iodide (PI) were purchased from Sigma (St. Louis, MO). The Annexin V-FITC apoptosis detection kit was purchased from Beijing 4A Biotech Co., Ltd. (Beijing, China). The antibodies against c-Myc, BRD4, Bcl-2, and BAX were obtained from Cell Signaling Technology Company (Beverly, MA), antibody against GAPDH was purchased from Chengdu Zen BioScience Co. Ltd. (Chengdu, China), and antibody against cyclin E and cyclin D1 were obtained from Abcam (Cambridge, MA, U.S.A.). The oligo primes used in RT-PCR were synthesized in Tsingke biological technology (Chengdu, China).

### Synthesis of compound YLT-LL-11

**Scheme 1:**
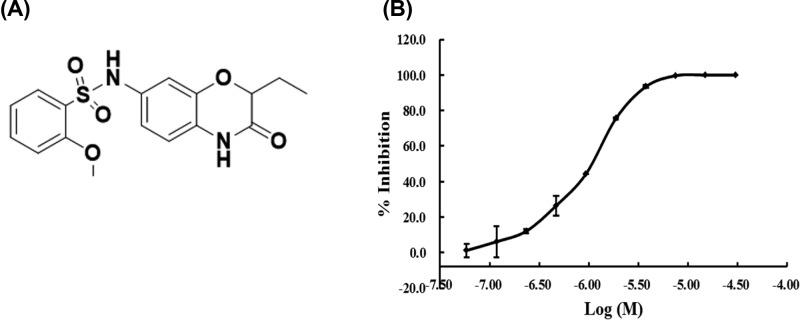
Synthetic route of YLT-LL-11 (**A**) K2CO3, DMF, 90°C, 8–12 h. (**B**) Fe, NH4CI, CH3CH2OH:H2O=3:1, reflux. (**C**) ArSO2CI, Triethylamine, Tetrahydrofuran, 0°C, Ar is methoxy phenyl group.

The compound YLT-LL-11 was synthesized according to Scheme 1 [22]. Briefly, 2-ethyl-7-nitro-2H-benzo[b] [1,4] oxazin-3(4H)-one (**3**) was prepared by cyclization of the 2-amino-5-nitrophenol (**1**) with ethyl 2-bromobutanoate (**2**). Reduction of the nitro group of **3** with iron powder and ammonium chloride at reflux temperature provides the corresponding aniline **4.** To a solution of **4** in tetrahydrofuran was added Et_3_N (3.0 eq) followed by 2-Methoxyphenylsulfonyl chloride (1.5 eq) at 0°C. The reaction mixture was allowed to react overnight at room temperature. The reaction mixture was evaporated and dissolved in 10 ml water. 1.0 N HCl was slowly added to the mixture (pH = 4), and the resulting solid was filtered and rinsed with water. The crude product was purified on a silica column to get N-(2-ethyl-3-oxo-3,4-dihydro-2H-benzo[b] [1,4] oxazin-7-yl)-2-methoxybenzenesulfonamide (**5, YLT-LL-11**) as brown solid. The structure of YLT-LL-11 was determined by ^1^H NMR, ^13^C NMR and MS (electrospray ionization (ESI)) as showed in the Supplementary Materials.

### Biochemical assay

Alphascreen™ assays were performed as previously reported [22,23]. Briefly, compound YLT-LL-11 was diluted in the recommended buffer (50 mM HEPES, pH 7.4). BRD4 (D1) protein (Biogenie, Canada) was added to microplate followed by nonbiotinylated peptide, solvent, or compound. After incubated at room temperature for 1 h, the plates were added acceptor and donor solution, and then incubated for another 1 h at room temperature under subdued light. Then, the plates were read on a PHERAstar FS plate reader (BMG Labtech, Germany). The data were fit using GraphPad Prism 5 to obtain IC_50_ value of YLT-LL-11 to BRD4 (D1).

### Cell culture

MV-4-11 (Peripheral blood B cell leukemia cells), OCI-LY10 (Diffuse large B cell lymphoma cell lines), RAMOS cell (Human B lymphocyte tumor cells), HEK293T (Human embryonic kidneys cells), COS-1 (Monkey fibroblast-like kidneys cells), MDA-MB-231 (Human epithelial breast cancer cells) were obtained from American Type Culture Collection (ATCC). The cells were cultured in RPIM 1640 or Dulbecco’s Modified Eagle’s Medium (DMEM) supplemented with 10% fetal bovine serum (Hyclone) and 1% Penicillin–Streptomycin Solution (100×, Hyclone) under humidified conditions with 5% CO_2_ at 37°C.

### MTT assays

The MTT assay was used to determine the respective cytotoxic effects of YLT-LL-11 on MV-4-11, OCI-LY10, RAMOS, HEK293T, COS-1, and MDA-MB-231 cell lines. Cells (1–3 × 10^4^/well) were seeded in 96-well culture plates, and treated with various concentrations of YLT-LL-11 or vehicle control (0.1% DMSO) for 48, 72, and 96 h. After treatment, 20 μl of a 5 mg/ml MTT solution was added to each well respectively and incubated for another 2–4 h at 37°C, then 50 μl of 20% SDS solution was added to each well and incubated at 37°C overnight (for HEK293T, COS-1, and MDA-MB-231 cell lines, the medium was discarded and 150 μl of DMSO was added to each well for 15–20 min). The absorbance was read at 570 nm by a Spectra MAX M5 microplate spectrophotometer (Molecular Devices, CA, U.S.A.). Each experiment was replicated at least three times.

### Apoptosis analysis

OCI-LY10 cells were cultured and exposed to DMSO and various concentrations of YLT-LL-11 for 72 h in cell incubator. Cells were then harvested and washed with phosphate buffer saline (PBS) three times, and resuspended in PBS. Cell suspensions were stained with Annexin V/FITC and PI according to the manufacturer’s instructions (Beijing 4A Biotech Co., Ltd). Briefly, PBS-washed cells were suspended in 100 μl FITC binding buffer at a minimum concentration of 1 × 10^6^ combined with 5 μl Annexin V/FITC for 15 min at 4°C. Then, 10 μl PI was added and analyzed by FCM. Cells that were Annexin V-negative and PI-negative were considered viable cells. Cells positive for Annexin V only were considered apoptotic, and cells positive for PI only were considered necrotic or late apoptotic. All samples were prepared in triplicate.

### Cell cycle analysis

Cell cycle distribution was determined by FCM with PI staining method. Briefly, OCI-LY10 cells were exposed to DMSO and various concentrations of YLT-LL-11 for 48 h in cell incubator. Then, the treated cells were harvested and fixed with 70% ice-cold ethanol overnight at 4°C. Control and treated cells were then washed and resuspended in PBS. Then incubated with PI staining solution (Sigma) and PBS at room temperature in the dark for 30 min and analyzed by FCM. All samples were prepared in triplicate. Data were analyzed with NovoExpress 1.1.2 software.

### Quantitative reverse transcriptase PCR

Total cellular mRNA was isolated using Trizol agent (Invitrogen), following the manufacturer’s instructions. The residual genomic DNA was wiped off prior to cDNA synthesis using PrimeScript™ RT reagent Kit with gDNA Eraser (Takara) on Gene amplification Machine (Gene Touch, BIOER, HangZhou, China) according to the manufacturer’s instructions. Quantitative reverse transcriptase PCR (qRT-PCR) was performed using 20 μl reaction volume containing 10 μl 2 × SYBR Premix Ex Taq II (SYBR^®^ Premix Ex Taq™ II; Takara), 6.4 μl distilled H_2_O, 0.8 μl forward primer, 0.8 μl reverse primer, and 2 μl of 1:10 diluted cDNA on the CFX96 Real-Time System (Bio-Rad). Each reaction was subjected to the following conditions: 95°C for 30 s, followed by 40 cycles of 95°C for 5 s, 60°C for 30 s, and 72°C for 30 s in 96-well optical reaction plates (Bio-Rad). The reference gene GAPDH was used to normalize the amplification of the target genes. Each qRT-PCR analysis was performed in triplicate. Primer sequences are listed in Supplementary Table S1.

### Western blot analysis

Western blotting analysis was performed as described previously [24]. Briefly, cells were incubated separately with DMSO (vehicle control) or indicated concentration of YLT-LL-11 for 4 days. Harvested cells were lysed in RIPA buffer (Beyotime, Beijing, China) on ice for 30 min and the cell lysates were centrifuged at 13000 ***g*** at 4°C for 15 min. The harvested protein lysates were equalized by BCA method before loading. After denatured in loading buffer, about 20–60 mg of total protein from each sample was separated according to the molecular weight on 12.5% sodium dodecyl sulfate/polyacrylamide (SDS/PAGE) gel and transferred onto polyvinylidene fluoride (PVDF) membranes (Merck Millipore, Massachusetts, U.S.A.). After blocked with 5% fat-free milk in TBS/T for 2 h at room temperature, the membranes were incubated in primary antibody overnight at 4°C. Then, the membranes were incubated with correspondent horseradish peroxidase-conjugated secondary antibodies [25]. The immunoreactive protein bands were detected using the enhanced chemiluminescence kit (Millipore, U.S.A.). A monoclonal GAPDH antibody was used as a control.

### Statistical analysis

Statistical analyses were carried out in Microsoft excel software. All the statistical data are expressed as mean ± SD for three independent experiments. In all statistical analysis, *, *P*<0.05; **, *P*<0.01; ***, *P*<0.001.

## Results

### Biochemical characterization of YLT-LL-11 as a potent inhibitor of BRD4

**Figure 1:**
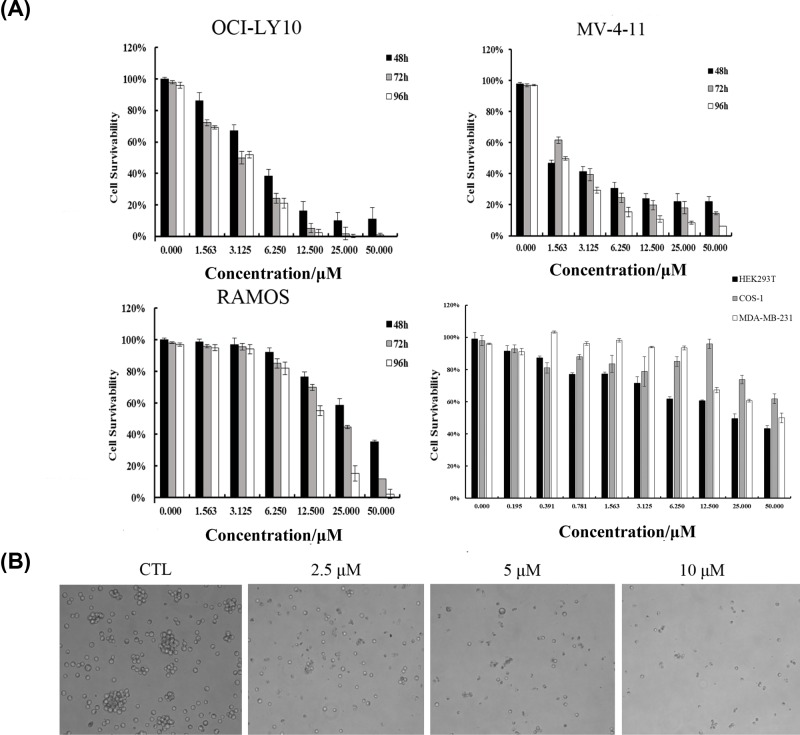
Structure and potency of YLT-LL-11 (**A**) The chemical structure of YLT-LL-11. (**B**) α-screen data of compound YLT-LL-11 against BRD4 (D1) protein. Each percentage of inhibition data were based on three independent titrations. The inhibition curve was fitted and shown as solid line to obtain the IC_50_ value of YLT-LL-11 to BRD4 (D1).

Our previous study verified benzomorpholinone derivatives as potential BET bromodomain inhibitors based on protein affinity and cell viability assays. Further optimization on this chemotype identified N-(2-ethyl-3-oxo-3,4-dihydro-2H-benzo[b] [1,4] oxazin-7-yl)-2-methoxybenzenesulfonamide (YLT-LL-11) as a promising candidate of BRD4 inhibitor. YLT-LL-11 was synthesized as Scheme 1. The inhibitory activity against BRD4 (D1) of YLT-LL-11 was evaluated using the AlphaScreen assay resulted in an IC_50_ of 999 nM (Figure 1), which is superior to our previously discovered benzomorpholinone derivatives. These results suggested that YLT-LL-11 is a potent inhibitor of BRD4.

### YLT-LL-11 inhibited the growth of cancer cells

**Figure 2:**
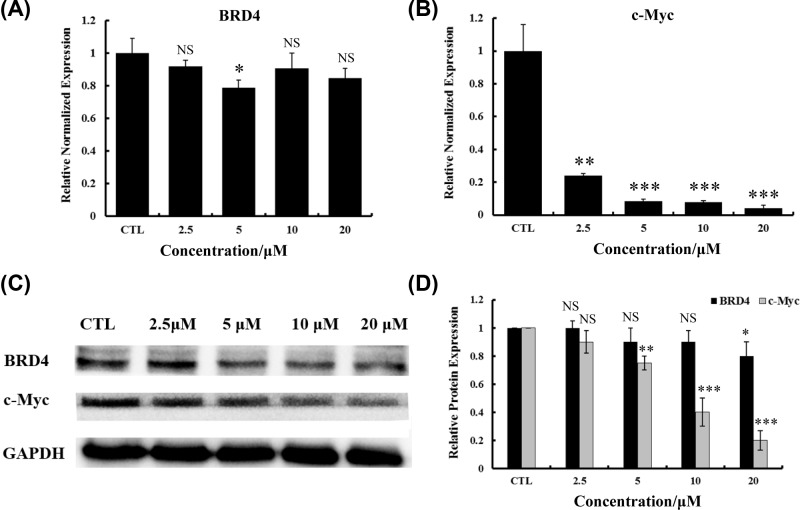
Effects of YLT-LL-11 on the cell growth and morphology in human hematologic malignancies cell lines (**A**) human hematologic malignancies cell lines OCI-LY10, MV-4-11, and RAMOS, were treated with different concentrations of YLT-LL-11 for 48, 72 or 96 h and cell viability was measured by MTT assays. Normal cell lines HEK293T and COS-1, BRD4 low expression cell lines MDA-MB-231 were treated with different concentrations of YLT-LL-11 for 96 h and cell viability was measured by MTT assays. Data are expressed as mean ± SD for three independent experiments. (**B**) Bright-field microscopy images showed the effect of different concentrations of YLT-LL-11 on cell morphology in OCI-LY10 cell line.

We next tested the ability of YLT-LL-11 to inhibit the growth of malignant hematopoietic cell lines using MTT assays. MV-4-11, OCI-LY10, and RAMOS were incubated with different concentrations of YLT-LL-11 for 48, 72 and 96 h. As showed in Figure 2A, YLT-LL-11 showed notable anti-proliferative activity against the MV-4-11 and OCI-LY10 cells in dose- and time-dependent manners. The growth rate of both MV-4-11 and OCI-LY10 cells lines was decreased obviously after 3 days of incubation, with an IC_50_ values of 2.1 and 3.2 μM, respectively. RAMOS cells were less sensitive to YLT-LL-11 in the MTT assays with an IC_50_ of 10 μM after 4 days of incubation. Moreover, we evaluated the cytotoxic effects of YLT-LL-11 on two normal cell lines HEK293T and COS-1. No obvious toxicities were found under the low concentrations. Furthermore, we also evaluated the anti-proliferation effect of YLT-LL-11 against a BRD4 low expression cell line MDA-MB-231 [26] to clarify the target specificity. The results showed that under the similar low concentrations, YLT-LL-11 showed no obvious inhibition of cell proliferation to MDA-MB-231 cell. Meanwhile, we found significant changes in cell morphlogy of OCI-LY10 as the compound concentrations increasing (Figure 2B), which encourage us to further investigate the role of BRD4 in DLBCL cells.

### YLT-LL-11 inhibited c-Myc transcription of OCI-LY10 cells

**Figure 3:**
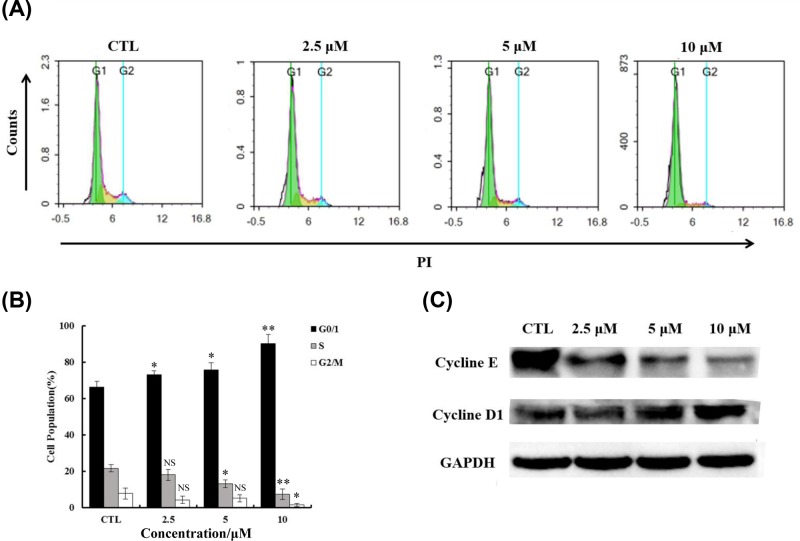
Intracellular effect on BRD4 and c-Myc transcription by YLT-LL-11 treatment Relative normalized expression of intracellular BRD4 (**A**) and c-Myc (**B**) in real-time PCR assays of OCI-LY10 cell line treated with YLT-LL-11 for 3 days. Data are expressed as mean ± SD for three independent experiments. (**C**) The protein expression levels of BRD4 and c-Myc via Western blotting assays. (**D**) Protein expression was qualified by the densitometry analysis using Image J. Statistical significance was assessed by unpaired *t* test. ^*^P<0.05, ^**^P<0.01, ^***^P<0.001.

The efficacy of BET inhibitors in several types of cancers is dependent on the down-regulation of MYC, an oncogene related to cell cycle progression, apoptosis, and cellular transformation [11,27,28]. To determine whether YLT-LL-11 attenuate the growth of DLBCL cells via MYC-dependent transcription, real-time PCR assays were performed to detect the changes in the transcription levels of both *BRD4* and c-*Myc*. As shown in Figure 3A,B, the relative normalized expression of *BRD4* was less influenced by different concentrations of YLT-LL-11, while a dose-dependent decrease of *c-Myc* was observed in the same assays. The c-*Myc* transcription level attenuated obviously when exposure to 5 μM of YLT-LL-11. The results were consistent with detected protein expression levels of BRD4 and c-Myc via Western blotting assays in YLT-LL-11-treated OCI-LY10 cells (Figure 3C,D). These results showed that exposure of cells with BRD4 inhibitor YLT-LL-11 resulted in a significant transcriptional down-regulation of c-Myc while the expression level of BRD4 was less influenced.

### YLT-LL-11 induced G0/G1 phase arrest of DLBCL cells

**Figure 4:**
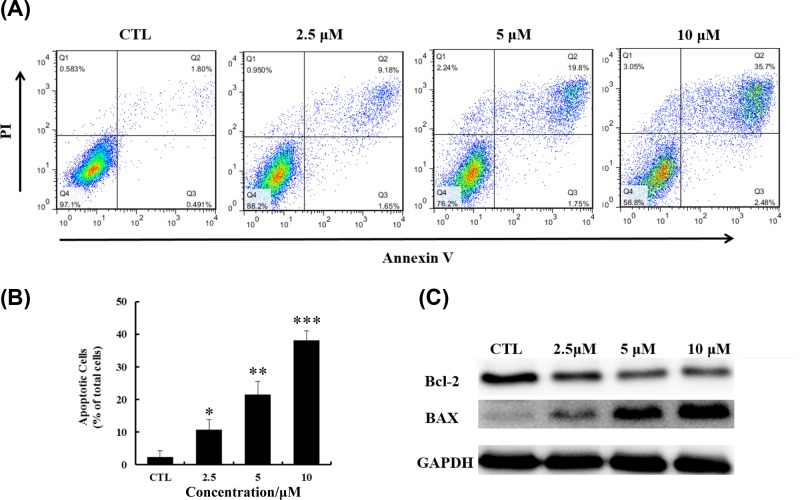
YLT-LL-11 induced G0/G1 cell cycle arrest of OCI-LY10 cell line (**A**) OCI-LY10 cells were treated with different concentrations of YLT-LL-11 for 3 days and the cell cycle attribution was analyzed by FCM after incubated with PI solution. (**B**) Quantified histograms display the effect of YLT-LL-11 on cell cycle distribution. Data are expressed as mean ± SD for three independent experiments. Statistical significance was assessed by unpaired *t* test. ^*^P<0.05, ^**^P<0.01, ^***^P<0.001. (**C**) The expression of cell cycle related protein cyclin E, cyclin D1 were evaluated by Western blotting.

To further understand the role of YLT-LL-11 in the inhibition of DLBCL cells proliferation, we performed cell cycle analysis by FCM (Figure 4A). In OCI-LY10 cells treated with different concentrations of YLT-LL-11 for 2 days, an accumulated percentage of cells from 66.45 to 90.37% in G0/G1 phase was detected in a concentration-dependent manner, which was accompanied by a decrease of cells in G2/M (from 21.69 to 7.41%) and S phases (from 7.84 to 1.52%) (Figure 4B). These results suggested that the anti-DLBCL effects of YLT-LL-11 might be associated with G0/G1 phase arrest, which is consistent with the effect of well-known BET inhibitors JQ1 in a panel of leukemia and lymphoma cell lines [27,29,30].

We then evaluated the expression of the cyclin-dependent kinases (CDKs) which related to cell cycle progression regulation. The Western blotting assays showed that the levels of cyclin E proteins decreased and cyclinD1 increased after treatment with YLT-LL-11 for 2 days, which further validated the cell cycle arrest when exposure to YLT-LL-11 (Figure 4C).

### YLT-LL-11 led to apoptosis in DLBCL cell

**Figure 5:**
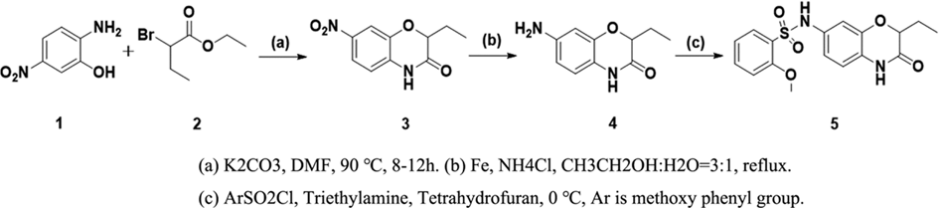
Treatment with YLT-LL-11 induced apoptosis of OCI-LY10 cell line (**A**) The apoptotic cell was analyzed using FCM after Annexin V/PI dual-labeling when treated with indicated concentrations of YLT-LL-11 for 3 days. The proportion of apoptotic cells (Q2 and Q3) are showed in the histograms (**B**). Data are expressed as mean ± SD for three independent experiments. Statistical significance was assessed by unpaired *t* test. ^*^P<0.05, ^**^P<0.01, ^***^P<0.001. (**C**) The level of apoptosis-related protein Bcl-2 and BAX were evaluated by Western blotting.

Cell morphology changes (Figure 2B) indicated that the apoptosis pathway might be activated in OCI-LY10 cells after YLT-LL-11 treatment. We investigated this mechanism by FCM and measuring the changes of intracellular apoptosis-related proteins by Western blotting (Figure 5). Annexin V/PI staining assays determined the pro-apoptotic effect of YLT-LL-11 on OCI-LY10 cells as showed in Figure 5A. After exposure of OCI-LY10 cells to YLT-LL-11 for 48 h, the early apoptotic cells (Q3) and the late apoptotic cells (Q2) remarkably increased from 10.8 to 38.2% as the concentration increased from 2.5 to 10 μM, whereas only 2.3% of apoptotic cells were detected in the vehicle group (Figure 5B). The data clearly indicated that YLT-LL-11 caused apoptosis of OCI-LY10 cells in a concentration-dependent manner. Moreover, an increased level of BAX and a decreased level of Bcl-2 were observed in the Western blotting assays (Figure 5C). Since BAX and Bcl-2 family members are the master regulators of apoptosis, these results further confirmed the apoptosis induced by YLT-LL-11.

## Discussion and conclusion

DLBCL is the most common type of B-cell NHL with significant biological heterogeneity which is difficult to treat with common chemotherapy regimen or targeted therapy. Novel therapeutic strategies are thus urgent needed to treat these clinical patients with relapsed or refractory DLBCL or patients who are resistant to all the current regimens.

A various of studies have proven BRD4 as a co-activator of MYC which plays significant role in cell proliferation especially in human malignant tumors. The transcription and expression of MYC in DLBCL cell lines were found to be down-regulated after BRD4 inhibition and resulted in cell growth inhibition, resembling the effects in other BRD4-dependent cancer cell lines, which suggested BRD4 would be a particularly compelling target for DLBCL treatment.

Through constantly optimizing the structure of benzomorpholinone derivatives, we got the compound YLT-LL-11 which has a powerful anti-cancer activity against human hematologic malignancies such as MV-4-11, OCI-LY10, and RAMOS. YLT-LL-11 showed favorable protein affinity to BRD4 and inhibited cellular MYC transcription and expression, thus exerted excellent inhibition effect of cell proliferation. The FCM analysis and Western blotting assays verified that YLT-LL-11 can inhibited cell cycle progression and further induced apoptosis of DLBCL cell line OCI-LY10. The compelling evidence indicated that YLT-LL-11 can act as a potential candidate targeting BRD4 for DLBCL treatment, which also verified benzomorpholinone as a biologically potent scaffold worthy further studies. However, further optimization toward benzomorpholinone scaffold are still needed to find better candidates with optimized potency and drug-like properties, such that suitable for evaluated *in vivo*.

Nowadays, the PROTACs (proteolysis-targeting chimera) technology has provided a novel therapeutic method via degradation of disease-associated proteins with small molecules [31–34]. PROTAC molecules can simultaneously binds E3 ubiquitin ligase and the target protein to cause ubiquitination and subsequent degradation of this target protein. The VHL- or cereblon-based PROTACs conjugating the pan-BET inhibitor motif JQ1 can cause sustained depletion of BRD4 [35–37]. More importantly, these BET PROTACs were found to be more potent in down-regulation of c-Myc and growth inhibition of cancer cell lines, as well as in tumor xenografts, which verified the efficacy and advantages of BET-PROTAC based technology as a novel approach to address the challenge of malignancies [38]. YLT-LL-11 can be also modified to PROTAC by conjugating an E3-ubiquitin ligase using a linker to exert potent BRD4 binding and degradation ability. And this modification of YLT-LL-11 may provide a path forward to promote the efficacy of this compound itself, but also afford YLT-LL-11 to achieve new therapeutic functions.

## Supplementary Material

Supplementary Table S1

## Abbreviations

BET: bromodomain and extra-terminal
CDKs: cyclin-dependent kinases
DLBCL: diffuse large B-cell lymphoma
DMSO: dimethyl sulfoxide
MTT: 3-(4, 5-Dimethylthiazol-2-yl)-2, 5-diphenyltetrazolium bromide
NHL: non-Hodgkin’s lymphoma
PBS: phosphate buffer saline
PI: propidium iodide
PROTAC: proteolysis-targeting chimera
qRT-PCR: quantitative reverse transcriptase PCR
