# Supplementary material for: A novel benzoxazinone derivative YLT-LL-11 inhibits diffuse large B-cell lymphoma growth via inducing cell cycle arrest and apoptosis
Source: Biosci Rep. 2019 Oct 11;39(10):BSR20190828. doi: 10.1042/BSR20190828 (PMC6822579; doi:10.1042/BSR20190828)
Supplement: Supplementary Table S1 [file BSR-2019-0828_supp.pdf]

Supplementary information

A novel benzoxazinone derivative YLT-LL-11 inhibits diffuse large B-cell lymphoma growth *via* inducing cell cycle arrest and apoptosis

Cuiting Peng[1,3 #], Changzhen Sun[2, #], Ningyu Wang[4], Yuanmin He[1], Jixiang Xu[1], Lanyang Gao[5], Yongqiong Deng[1], Jianqiao Zhong[1], Xia Xiong[1], Li Liu[1, *]

[1] Department of dermatology, The affiliated hospital of Southwest Medical University, Luzhou, China.

[2] Drug Research Center of Integrated Traditional Chinese and Western Medicine, Affiliated Traditional Chinese Medicine Hospital, Southwest Medical University, Luzhou, China.

[3] Department of Obstetrics& Gynecology, West China Second University Hospital, Sichuan University, Chengdu, China.

[4] School of Life Science and Engineering, Southwest Jiaotong University, Chengdu, China.

[5] Department of Science and Technology, The affiliated hospital of Southwest Medical University, Luzhou, China.

[#] These authors contributed equally to this study.

[*] Address correspondence to Li Liu: liuli@swmu.edu.cn

The structure of the YLT-LL-11 was characterized by nuclear magnetic resonance (NMR) and mass spectrometry (MS).

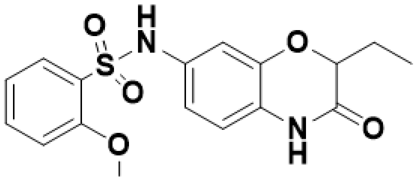

^{1}H NMR (400 MHz, DMSO-d_6) δ 10.49 (s, 1H), 9.80 (s, 1H), 7.71 (dd, J = 7.8, 1.7 Hz, 1H), 7.59 – 7.52 (m, 1H), 7.16 (d, J = 8.3 Hz, 1H), 7.01 (t, J = 7.6 Hz, 1H), 6.67 (d, J = 4.4 Hz, 3H), 4.41 (dd, J = 7.8, 4.5 Hz, 1H), 3.89 (s, 3H), 1.74 (m, J = 7.4, 4.5 Hz, 1H), 1.65 (m, J = 14.5, 7.4 Hz, 1H), 0.91 (t, J = 7.4 Hz, 3H).

^{13}C NMR (101 MHz, DMSO-d_6) δ 166.27, 156.79, 143.01, 135.48, 133.48, 130.75, 126.66, 124.10, 120.51, 115.95, 114.57, 113.22, 109.16, 77.55, 56.54, 23.67, 9.51.

Mass spectrometry (ESI, *m/z*): 361.0869 $[M-H]^-$.

Supplementary Table 1 Primers used in real-time PCR

**Application**	**sequences (5’ to 3’)**
c-Myc-forward	GCCACGTCTCCACACATCAG
c-Myc-reverse	TCTTGGCAGCAGGATAGTCCTT
BRD4- forward	ACCTCCAACCCTAACAAGCC
BRD4-reverse	TTTCCATAGTGTCTTGAGCACC
GAPDH- forward	GCGATGCTGGCGCTGAGTACGTCG
GAPDH-reverse	GGGCATCAGCAGAGGGGGCAGAGA
